# Basal Rot of *Narcissus*: Understanding Pathogenicity in *Fusarium oxysporum* f. sp. *narcissi*

**DOI:** 10.3389/fmicb.2019.02905

**Published:** 2019-12-19

**Authors:** Andrew Taylor, Andrew D. Armitage, Claire Handy, Alison C. Jackson, Michelle T. Hulin, Richard J. Harrison, John P. Clarkson

**Affiliations:** ^1^Warwick Crop Centre, School of Life Sciences, University of Warwick, Warwick, United Kingdom; ^2^NIAB EMR, East Malling, United Kingdom

**Keywords:** *Fusarium oxysporum* f. sp. *narcissi*, *Narcissus*, daffodil, basal rot, pathogenicity, *Secreted in Xylem*

## Abstract

*Fusarium oxysporum* is a globally distributed soilborne fungal pathogen causing root rots, bulb rots, crown rots and vascular wilts on a range of horticultural plants. Pathogenic *F. oxysporum* isolates are highly host specific and are classified as *formae speciales*. *Narcissus* is an important ornamental crop and both the quality and yield of flowers and bulbs can be severely affected by a basal rot caused by *F. oxysporum* f. sp. *narcissi* (FON); 154 *Fusarium* isolates were obtained from different locations and *Narcissus* cultivars in the United Kingdom, representing a valuable resource. A subset of 30 *F. oxysporum* isolates were all found to be pathogenic and were therefore identified as FON. Molecular characterisation of isolates through sequencing of three housekeeping genes, suggested a monophyletic origin with little divergence. PCR detection of 14 *Secreted in Xylem* (*SIX*) genes, previously shown to be associated with pathogenicity in other *F. oxysporum* f. spp., revealed different complements of *SIX7*, *SIX9*, *SIX10*, *SIX12* and *SIX13* within FON isolates which may suggest a race structure. *SIX* gene sequences were unique to FON and *SIX10* was present in all isolates, allowing for molecular identification of FON for the first time. The genome of a highly pathogenic isolate was sequenced and lineage specific (LS) regions identified which harboured putative effectors including the *SIX* genes. Real-time RT-PCR, showed that *SIX* genes and selected putative effectors were expressed *in planta* with many significantly upregulated during infection. This is the first study to characterise molecular variation in FON and provide an analysis of the FON genome. Identification of expressed genes potentially associated with virulence provides the basis for future functional studies and new targets for molecular diagnostics.

## Introduction

*Fusarium oxysporum* is a globally important plant pathogen, causing root rots, bulb rots, crown rots and vascular wilts on a wide range of horticultural crops and ornamental plants including onion, lettuce, tomato, cucumber, pea, daffodil (*Narcissus*), carnation, lily, tulips and *Gladiolus* (Leslie and Summerell, 2006; Michielse and Rep, 2009). Pathogenic *F. oxysporum* isolates are highly host specific and are therefore classified as *formae speciales* (Leslie and Summerell, 2006; Michielse and Rep, 2009). There are now 106 well-characterised *formae speciales*, 37 with insufficient evidence to definitively classify and 58 plant species susceptible to *F. oxysporum* that do not have a defined *forma specialis* (Edel-Hermann and Lecomte, 2019). Recent research on *F. oxysporum* f. sp. *lycopersici* (FOL, infecting tomato) has led to the discovery that host specific virulence is associated with lineage specific (LS) chromosomes; for instance, it has been demonstrated that a non-pathogenic *F. oxysporum* isolate can acquire pathogenicity against tomato by transfer of an LS chromosome (Ma et al., 2010; Rep and Kistler, 2010; Ma et al., 2015). LS chromosomes have a low gene density (after transposons are taken into account) and are enriched for small secreted proteins (Ma et al., 2010; Schmidt et al., 2013; Armitage et al., 2018) which include those encoded by *Secreted in Xylem* (*SIX*) genes, 14 of which have been identified in FOL (Houterman et al., 2009; Schmidt et al., 2013). Functional studies have shown that *SIX1*, *SIX3*, *SIX5* and *SIX6* have a direct role in FOL virulence (Rep, 2005; Houterman et al., 2009; Takken and Rep, 2010; Gawehns et al., 2014; Ma et al., 2015); however, *SIX* genes show little or no homology to each other or any other genes of known function. Several studies have identified *SIX* gene homologues in a range of *F. oxysporum* f. spp. and their presence is very often associated with pathogenicity (Lievens et al., 2009; Fraser-Smith et al., 2014; Taylor et al., 2016; van Dam et al., 2016). In addition, members of nine transcription factor families on FOL LS regions (TF1-9) have roles in virulence through controlling effector gene expression (van der Does et al., 2016). In particular, research investigating *F. oxysporum* f. sp. *phaseoli*, causing wilt of common bean, revealed that *Fusarium transcription factor* (*FTF1*) from the TF1 gene family, plays a role in virulence through controlling the expression of at least two *SIX* genes (Nino-Sanchez et al., 2016).

Daffodil (*Narcissus* spp.) is one of the most widely cultivated ornamental bulb crops of temperate regions. The major production areas are the United Kingdom (3808 ha per annum), Netherlands (1756 ha) and United States (410 ha) although smaller areas are cultivated across the world (Hanks, 2013). In the United Kingdom, bulbs are particularly prone to infection by soil-borne pathogens due to the standard biennial growing system employed whereby after 2 years in the field, bulbs are lifted and used as a replanting stock (Hanks, 2013). One of the most economically damaging pathogens of *Narcissus* is *F. oxysporum* f. sp. *narcissi* (FON) Snyder and Hansen, which infects the roots or damaged basal plates resulting in soft and rotting bulbs (Linfield, 1994). Affected bulbs do not sprout, or produce short-lived shoots, or chlorotic, prematurely senescing foliage with few or no flowers. Infection can occur in the field or in storage (Hanks, 1996). Although FON infection is favoured by higher temperatures, a minimum temperature range of 8–10°C has been reported for germination of chlamydospores and growth of mycelium (Price, 1977) with infection optimum at 29°C (McClennan, 1952).

Control of FON is challenging, mainly due to the production of chlamydospores which can survive in the soil for many years. Under laboratory conditions, chlamydospores of a *F. oxysporum* isolate from muskmelon were shown to be viable for at least 17 years (McKeen and Wensley, 1961). In the United Kingdom, daffodil bulbs which are lifted from the soil are routinely subjected to hot water treatment (44.4°C for 3 h; Qiu et al., 1993; Hanks and Linfield, 1999) during which bulbs can become infected with FON (Hawker, 1935). Until the late 2000s, formalin was also added to the hot water tank to enhance nematode control and to kill FON chlamydospores (Linfield, 1994; Hanks and Linfield, 1999). Subsequently, formalin has been replaced by fungicides such as thiabendazole and chlorothalonil (Hanks, 1996). There is some evidence that dipping bulbs in a biological control agent such as Streptomyces, as part of an integrated control approach, can help reduce the incidence of basal rot but this is an area which requires more research (Hiltunen et al., 1995). Whilst some partial resistance to FON has been identified, particularly in material related to the cv. St. Keverne and also in some related wild germplasm (Nicholson et al., 1989; Bowes et al., 1992; Linfield, 1992), the genetics of this resistance have not been investigated. It has been proposed that the mechanism of resistance in St. Keverne is controlled by multiple factors, including the accumulation of antifungal compounds and cell wall modification (Nicholson et al., 1989).

Whilst differences in pathogenicity and morphology between FON isolates has been occasionally reported (Beale and Pitt, 1989; Linfield, 1994), these studies were conducted more than 20 years ago and as such may not represent the current diversity of FON isolates. No research has specifically characterised isolates based on DNA sequence or attempted to identify genes potentially associated with FON virulence. This study assembled a contemporary collection of FON isolates from different United Kingdom *Narcissus* cultivars and locations and characterised them through PCR and sequencing of housekeeping and putative effector genes. Genome sequencing of a single FON isolate was carried out to allow comparative analysis with FOL and *F. oxysporum* f. sp. *cepae* (FOC), which causes a basal rot of onion bulbs, including identification of putative LS effector candidates. The expression of putative FON effectors *in planta* was also examined using real-time RT-PCR.

## Materials and Methods

### Collection of *Fusarium* Isolates From *Narcissus* and Pathogenicity Testing

*Narcissus* bulb samples with symptoms of basal rot representing 30 cultivars were obtained from 39 different locations in the United Kingdom in 2012 (Supplementary Table S1). Five bulbs from each sample were split lengthways and isolations carried out from internal infected scales as previously described for onion (Taylor et al., 2016). Fungal colonies identified as *Fusarium* were assigned to one of eight groups based on their morphology (colour/growth habit) on 50% potato dextrose agar (PDA). Isolates were placed in medium- and long-term storage on PDA slopes (4°C) and as spore suspensions in potato dextrose broth containing 20% glycerol (−80°C), respectively. A subset of 30 *Fusarium* isolates from the different morphology groups were selected for further analysis based on their relative frequencies and geographic origin (Table 1).

**TABLE 1:** Lesion size on *Narcissus* bulbs, culture morphology group and presence/absence of putative effector genes (*SIX*, BFJ63_5681, BFJ63_18062, BFJ63_18633) and *Fusarium transcription factor* (*FTF*) genes (as determined by PCR) for 30 FON isolates from different *Narcissus* cultivars and locations.

**Isolate**	**Morphology**	**Location**	**Cultivar**	**Lesion area (cm^2^)**	***SIX*7**	***SIX*9**	***SIX*10**	***SIX*12**	***SIX*13**	**18062**	**5681**	**18633**	***FTF1***	***FTF2***
FON139	4	Falmouth, Cornwall	Scrumpy	8.28	+	+	+	+	–	+	+	+	A	+
FON81	4	Truro, Cornwall	Salome	7.49	–	–	+	–	–	+	+	+	–	+
FON63	5	Truro, Cornwall	Magnificence	7.37	+	+	+	+	+	+	+	+	B	+
FON115	4	Falmouth, Cornwall	Pinza	7.22	+	+	+	+	–	+	+	+	–	+
FON46	4	Truro, Cornwall	Salome	7.05	–	–	+	–	–	+	+	+	–	+
FON7	7	Norfolk	White Lion	6.84	+	+	+	+	–	+	+	+	A	+
FON42	1	Holt, Norfolk	Golden Ducat	6.81	+	+	+	+	–	+	+	+	A	+
FON75	4	Norwich, Norfolk	Pheasant’s Eye	6.79	+	+	+	+	–	+	+	+	–	+
FON3	4	Norfolk	White Lion	6.71	–	–	+	–	–	+	+	+	–	+
FON89	4	Cornwall	Jedna	6.54	+	+	+	+	–	+	+	+	B	+
FON97	2	Cornwall	Hollywood	6.42	+	+	+	+	–	+	+	+	A	+
FON122	7	Falmouth, Cornwall	Unique	6.4	+	+	+	+	–	+	+	+	A	+
FON24	3	Boston, Lincs	Carlton	6.36	+	+	+	+	–	+	+	+	B	+
FON29	8	Norfolk	Fortune	6.18	+	+	+	+	–	+	+	+	A	+
FON11	7	E Cornwall	Carlton	6.12	+	+	+	+	–	+	+	+	A	+
FON38	2	Spalding, Lincs	Carlton	5.86	+	+	+	+	–	+	+	+	B	+
FON34	4	Holt, Norfolk	Sempre Avanti	5.78	+	+	+	+	–	+	+	+	A	+
FON94	7	Cornwall	Hollywood	5.53	+	+	+	+	–	+	+	+	A	+
FON 87	6	Cornwall	Golden Ducat	5.36	+	+	+	+	–	+	+	+	B	+
FON118	1	Falmouth, Cornwall	Orkney	4.59	+	+	+	+	–	+	+	+	A	+
FON77	1	Norwich, Norfolk	Pheasant’s Eye	4.44	–	+	+	–	–	+	+	+	B	+
FON55	7	Penzance	White Lion	4.44	+	+	+	+	–	+	+	+	B	+
FON58	2	Spalding, Lincs	Great Leap	4.15	+	+	+	+	–	+	+	+	A	+
FON141	1	Falmouth, Cornwall	Mithrel	4.02	+	+	+	+	–	+	+	+	B	+
FON129	6	Falmouth, Cornwall	Whiskey Galore	4.02	+	+	+	+	–	+	+	+	A	+
FON19	1	Spalding, Lincs	Quirinus	3.64	+	+	+	+	–	+	+	+	B	+
FON15	4	Spalding, Lincs	St Keverne	3.53	+	+	+	+	–	+	+	+	A	+
FON152	4	Moulton, Lincs	Carlton	3.36	+	+	+	+	–	+	+	+	–	+
FON133	7	Falmouth, Cornwall	Hampton Court	3.3	+	+	+	+	–	+	+	+	A	+
FON68	7	Spalding, Lincs	Spellbinder	3.24	+	+	+	+	–	+	+	+	B	+

**TABLE 2:** Genomic statistics for FON139 and reference genomes for FOL, FOC and FO.

**Organism**	**FON**	**FOL**	**FOC**	**FO**
**Isolate**	**139**	**4287**	**Fus2**	**fo47**
**(A) Assembly stats:**				
Assembly size (Mb)	57.5	61.5	53.4	49.7
Contigs	4349	15 +73^∗^	34	124
Largest contig (kb)	1164	6855	6434	6199
N50 (kb)	181	4590	414	3844
Kb Repeatmasked	5690	10,092	5632	2819
% Repeatmasked	9.89	16.42	10.54	5.68
Sodariomycete genes (BUSCO)	3684	3599	3687	3687
% Sodariomycete genes (BUSCO)	98.9	96.6	99.0	99.0
**(B) Gene models:**				
Total genes	20493	20925	18855	18191
Total proteins	20701	27347	19371	24818
*Braker transcripts*	19059	–	–	–
*CodingQuarry transcripts*	1642	–	–	–
Sodariomycete genes (BUSCO)	3666	3577	3668	3687
% Sodariomycete genes (BUSCO)	98.4	96.0	98.5	99.0
Secreted genes	1560	1493	1449	1409
**(C) Effector-associated *mimp* motif:**				
*Mimps* in genome	207	158	153	25
Genes in 2 kb of *mimp*	119	108	155	24
Secreted genes in 2 kb of *mimp*	29	22	31	3
**(D) Effector candidates:**				
Secreted and effector-like structure	407	351	355	291
Secreted CAZYmes	399	386	386	382

*Summarised assembly **(A)** and gene model **(B)** statistics are shown, as well as results of searches for pathogenicity-associated miniature impala (*mimp*) sequences **(C)** and identification of effector candidates **(D)**. Gene models predicted in this study show values predicted by Braker and additional genes predicted by CodingQuarry. ^∗^FOL chromosomes + non-scaffolded contigs.*

Isolates were tested for pathogenicity on *Narcissus* bulbs of the susceptible cultivar “Carlton.” Agar plugs (5 mm diameter) from actively growing colonies of each isolate were used to inoculate five replicate “bulb units” prepared by peeling away the outer scales (Supplementary Figure S1). Sterile agar plugs were used as a control and the non-pathogenic isolate Fo47 (Aimé et al., 2013) was also included. Inoculated bulb units were placed on damp tissue in a sealed container and incubated at 25°C. Lesions were photographed at 7, 12, 15 and 20 days after inoculation and the area of each lesion calculated at each time point using ImageJ software (Schneider et al., 2012). The experiment was repeated once. The significance of differences in lesion sizes at 20 dpi was assessed by ANOVA using Genstat version 18 (VSN International).

### Molecular Characterisation of *Fusarium* Isolates From *Narcissus*

DNA was extracted from freeze-dried mycelium of each isolate using a DNeasy Mini Kit (Qiagen) and PCR amplification carried out for three housekeeping genes using previously published primers and cycling conditions (Taylor et al., 2016; Supplementary Table S2); *translation elongation factor 1 alpha* (*EF-α*, exTEF-F/FUexTEF-R primers), *β-tubulin* (*TUB2*, T1/T22 primers) and *RNA polymerase II second largest subunit* (*RPB2*, 7cF/11aR primers). PCR products were purified using a QIAquick PCR Purification Kit (Qiagen) and then sequenced using the forward primer (Eurofins-GATC). *Fusarium* isolates were identified to species level based on *EF-α* sequence using BLAST searches (Boratyn et al., 2013). Sequences of all three genes were concatenated and aligned (CLUSTALW method) using MEGA version 7 (Kumar et al., 2016) and a maximum likelihood tree constructed using the gamma distributed Kimura two-parameter model (Kimura, 1980). Sequences from selected *F. oxysporum* f. spp. were also included for comparison and the tree rooted using *Fusarium proliferatum*.

### Identification and Sequencing of *SIX* Genes, Transcription Factors and Putative Novel Effectors

The 30 *Fusarium* isolates from *Narcissus* were screened for the presence of 14 *SIX* genes by PCR using previously published primers and thermocycling conditions designed for both FOC (Taylor et al., 2016) and FOL (Lievens et al., 2009). Where PCR amplicons were obtained, this was confirmed by an additional (conventional) PCR using published primers designed for real-time PCR (Supplementary Table S2, data not shown). Primers were also designed to amplify *FTF1* and *FTF2*, based on the genome sequence of isolate FON139 (described below); FON *FTF1*-F (5′-GGGTTGAATCTCACGTATCCTGC-3′)/FON *FTF1*-R (5′-TCCATTCGAGCCCTGCCCAAAG-3′) and FON *FTF2*-F (5′-CGGTCAAGCAATTCGCATGGC-3′/FON *FTF2*-R (5′-GTTCTCTGTCTTGTGGACGTCG-3′). Primers were used at a final concentration of 0.5 μM each in a 20 μl reaction following the same conditions outlined for *SIX* gene detection in FOC (Taylor et al., 2016) but with an annealing temperature of 65°C for *FTF1* and 63°C for *FTF2*. PCR amplicons for *FTF1* were purified and sequenced as described previously and sequences aligned (CLUSTALW method) and compared using MEGA version 7 (Kumar et al., 2016). Finally, all 30 FON isolates were screened for the presence of three other putative effectors identified by the presence of a *mimp* motif from genome analysis (Table 3, described below), BFJ63_18062 (FON18062 F: 5′-ATGGCGAACTGGTCTTGGCTCC-3′/FON18062 R” 5′-CTTGAGCACCCCACGGAACACT-3′); BFJ63_5681 (FON 5681 F: 5′-CCGTGTTCCTTGCTTTTACTGCCG-3′/FON5681 R: 5′-TGCAGCCGCCATCCTTGTAGTAC-3′) and BFJ63_18633 (FON18633 F: 5′-CCAGGCTTTTCCTCGAACCGCA-3′/FON 18633 R: 5′-TCGACGCTGTAGTCCGCAAAGAG-3′), following the same reaction set-up and cycling conditions outlined for *SIX* gene detection in FOC (Taylor et al., 2016) but with an annealing temperature of 65°C.

**TABLE 3:** Details of *SIX* gene homologs and an additional 27 genes in the FON genome located within 2 kb of *mimps* and encoding predicted secreted proteins (Sec + *mimp*).

**FON gene ID**	**FON contig**	**Sec. + *mimp***	**Summarised effector function**	**Orthogroup**	**Orthogroup contents**
**BFJ63_17906**	**Contig_728 (LS)**	No	***SIX*7 homolog | Transmembrane domain**	**Orthogroup13453**	**FON(1):FOL(0):FOC(1):FO(0)**
**BFJ63_19066**	**Contig_1485 (LS)**	Yes	***SIX*9 homolog | EffP**	**Orthogroup5087**	**FON(1):FOL(2):FOC(2):FO(0)**
**BFJ63_17905**	**Contig_728 (LS)**	Yes	***SIX*10 homolog | EffP**	**Orthogroup6913**	**FON(2):FOL(1):FOC(1):FO(0)**
**BFJ63_17909**	**Contig_728 (LS)**	No	***SIX*12 homolog**	**Orthogroup13592**	**FON(1):FOL(0):FOC(1):FO(0)**
BFJ63_3347	Contig_16 (C)	Yes	CAZY | IPR017853;Glycoside hydrolase superfamily	Orthogroup6617	FON(1):FOL(1):FOC(1):FO(1)
**BFJ63_5681**	**Contig_32 (C)**	Yes	**EffP | IPR002532;Hantavirus glycoprotein G2**	**Orthogroup11209**	**FON(1):FOL(1):FOC(1):FO(1)**
BFJ63_17239	Contig_492 (LS)	Yes	IPR031348;Fungal N-terminal domain of STAND protein	Orthogroup4168	FON(2):FOL(0):FOC(2):FO(1)
BFJ63_17356	Contig_522 (LS)	Yes	IPR005152;Lipase, secreted	Orthogroup1532	FON(5):FOL(1):FOC(2):FO(1)
BFJ63_17513	Contig_579 (LS)	Yes	EffP	Orthogroup13095	FON(2):FOL(0):FOC(0):FO(0)
BFJ63_17710	Contig_648 (LS)	Yes	PF12697;Alpha/beta hydrolase family	Orthogroup3054	FON(3):FOL(1):FOC(1):FO(1)
BFJ63_17810	Contig_686 (LS)	Yes	CAZY | IPR011050;Pectin lyase fold/virulence factor	Orthogroup299	FON(8):FOL(6):FOC(4):FO(5)
BFJ63_18064	Contig_796 (LS)	Yes	EffP	Singleton	–
**BFJ63_18062**	**Contig_796 (LS)**	Yes	**EffP**	**Orthogroup13571**	**FON(1):FOL(0):FOC(1):FO(0)**
BFJ63_18183	Contig_848 (LS)	Yes	EffP	Orthogroup13372	FON(1):FOL(0):FOC(1):FO(0)
BFJ63_18181	Contig_848 (LS)	Yes	EffP	Orthogroup13378	FON(1):FOL(0):FOC(1):FO(0)
BFJ63_18368	Contig_947 (LS)	Yes	CAZY | IPR011050;Pectin lyase fold/virulence factor	Orthogroup2534	FON(2):FOL(1):FOC(3):FO(1)
BFJ63_18450	Contig_995 (LS)	Yes	IPR001563;Peptidase S10, serine carboxypeptidase	Orthogroup1545	FON(3):FOL(2):FOC(2):FO(2)
BFJ63_18534	Contig_1045 (LS)	Yes	EffP | IPR032710;NTF2-like domain	Orthogroup13078	FON(2):FOL(0):FOC(0):FO(0)
BFJ63_18557	Contig_1059 (LS)	Yes	EffP	Orthogroup13112	FON(2):FOL(0):FOC(0):FO(0)
**BFJ63_18633**	**Contig_1107 (LS)**	Yes	**EffP**	**Singleton**	–
BFJ63_18752	Contig_1194 (LS)	Yes	IPR001506;Peptidase M12A, astacin	Orthogroup12558	FON(1):FOL(0):FOC(2):FO(0)
BFJ63_18778	Contig_1221 (LS)	Yes	–	Singleton	–
BFJ63_18926	Contig_1352 (LS)	Yes	EffP	Orthogroup13360	FON(1):FOL(0):FOC(1):FO(0)
BFJ63_18986	Contig_1408 (LS)	Yes	EffP | G3DSA:2.10.80.10	Orthogroup4560	FON(4):FOL(0):FOC(1):FO(0)
BFJ63_18985	Contig_1408 (LS)	Yes	EffP	Orthogroup13095	FON(2):FOL(0):FOC(0):FO(0)
BFJ63_19093	Contig_1508 (LS)	Yes	EffP	Orthogroup13113	FON(2):FOL(0):FOC(0):FO(0)
BFJ63_19121	Contig_1536 (LS)	Yes	EffP	Singleton	–
BFJ63_19127	Contig_1542 (LS)	Yes	EffP | G3DSA:2.10.80.10;	Orthogroup4560	FON(4):FOL(0):FOC(1):FO(0)
BFJ63_19143	Contig_1559 (LS)	Yes	EffP	Orthogroup13098	FON(2):FOL(0):FOC(0):FO(0)
BFJ63_19153	Contig_1568 (LS)	Yes	EffP	Orthogroup13114	FON(2):FOL(0):FOC(0):FO(0)
BFJ63_19631	Contig_2189 (LS)	Yes	–	Orthogroup13584	FON(1):FOL(0):FOC(1):FO(0)

*Genomic location within the FON assembly is shown including whether the contig was identified as core (C) or lineage specific (LS). Summaries of Interproscan annotations are provided along with indication of whether the gene is identified as encoding a protein with an effector-like structure (EffP) or carbohydrate active enzyme (CAZY). The orthology group is shown for each gene as well as the number of proteins assigned to that orthogroup from each of the genomes (FON, FOC, FOL, FO) used in the analysis. Genes analysed for their expression *in planta* are in bold.*

### Expression of FON *SIX* Genes and Other Putative Effectors *in planta*

Dried scales were removed from *Narcissus* bulbs (cv. Carlton) and inner scales separated to obtain 1–2 mm thick sections. These were then cut into 2 × 2 cm squares and surface sterilised by immersing in 70% ethanol for 2 min before rinsing twice in sterile distilled water. Scale sections were inoculated by placing a 5 mm agar plug removed from the edge of an actively growing colony of FON63 in the centre, mycelium side down. Scales were placed on moist chromatography paper (Whatman 3MM, Fisher Scientific, United Kingdom) in clear plastic boxes with lids (three per box) and incubated at 25°C in the dark. Four replicate samples of infected scales were taken each day from 0 (pre-inoculation) to 6 dpi by removing a 10 mm section around the inoculation point with a cork borer. Agar plugs were then removed and the infected *Narcissus* tissue samples flash frozen in liquid N before storing at −80°C. Mock inoculations were set up using plugs of PDA. RNA was extracted using the Spectrum plant total RNA extraction kit (Sigma, United Kingdom) following the manufacturers guidelines for a starchy plant storage organ. DNA was removed using DNase I (Sigma, United Kingdom) and first strand cDNA synthesised from 300 ng of RNA using Superscript II reverse transcriptase (Life Technologies). The expression of five *SIX* genes (*SIX7, SIX9, SIX10, SIX12, SIX13*), three other putative effectors (BFJ63_5681, BFJ63_18062, BFJ63_18633; Table 3) and two housekeeping genes (*EF1α* and *TUB2*) was then determined using previously published primers and cycling conditions (Taylor et al., 2016) with the exception of *SIX13* where new primers were designed (5′-ACAGCACGGGACAGCTTACA-3′/5′-CGTCAGAGGGGTAGCCACAT-3′, annealing temperature 60 °C). Primers for the three putative effectors were as described in the previous section. Quantitative PCR was carried out using a Roche Lightcycler 480 and expression determined using a standard curve method and normalised to the geometric mean of *EF1α* and *TUB2*. The statistical significance of differences in relative expression compared to that at 1 dpi was determined using paired, two-tailed *t*-tests.

### FON Genome Sequencing, Assembly, Gene Prediction and Orthology Analysis

DNA was extracted from freeze-dried mycelium of FON139 using the GenElute Plant Genomic DNA Miniprep Kit (Sigma) and genomic libraries prepared using the TruSeq DNA Sample Prep Kit (Illumina), following the manufacturer’s Low Sample Protocol. Libraries were sequenced using Illumina Miseq v3 2 × 300 bp PE (MS-102-3003). *De novo* assembly was performed using Spades v.3.5.0 (Nurk et al., 2013) with assembly quality statistics summarised using Quast (Gurevich et al., 2013). Single copy core Sodariomycete genes were identified using BUSCO v3 and used to assess assembly completeness (Simão et al., 2015). RepeatModeler, RepeatMasker and transposonPSI were used to identify repetitive and low complexity regions^1^,^2^. Gene prediction was performed on softmasked genomes using Braker1 v.2 (Hoff et al., 2016, fungal flag), a pipeline for automated training and gene prediction of AUGUSTUS 3.1 (Stanke and Morgenstern, 2005). Additional gene models were called in intergenic regions using CodingQuarry v.2 (Testa et al., 2015, pathogen flag). RNAseq data generated from FOC and the non-pathogenic Fo47 (FO, Armitage et al., 2018) were aligned to the FON genome using STAR (Dobin et al., 2013), and used in the training of Braker and CodingQuarry gene models for FON.

Orthology was identified between predicted FON proteins and those in the publicly available genomes for the non-pathogenic *F. oxysporum* isolate Fo47, FOC isolate Fus2 and FOL isolate 4287 (Ma et al., 2010; Armitage et al., 2018). OrthoMCL v.2.0.9 (Li et al., 2003) was run with an inflation value of 5 on the combined set of predicted proteins. Venn diagrams visualising genes common between proteomes were plotted using the R package VennDiagram (Chen and Boutros, 2011). Genes in orthogroups containing a single gene from both FON and FOL were used to assess synteny between genomes. Genes in a 1:1 relationship were used visualise synteny between FON contigs and FOL chromosomes with plots generated with Circos v.0.69 (Krzywinski et al., 2009).

Draft functional annotations were determined for gene models using InterProScan-5.18-57.0 (Jones et al., 2014) and through identifying homology between predicted proteins and those contained in the July 2016 release of the SwissProt database (Bairoch and Apweiler, 2000) using BLASTP (E-value > 1 × 10^–100^). Putative secreted proteins were identified through prediction of signal peptides using SignalP v.4.1 and removing those predicted to contain transmembrane domains using TMHMM v.2.0 (Krogh et al., 2001; Petersen et al., 2011). EffectorP v1.0 was used to screen secreted proteins for characteristics of length, net charge and amino acid content typical of fungal effectors (Sperschneider et al., 2016). Secreted proteins were also screened for carbohydrate active enzymes using HMM models from the dbCAN database (Huang et al., 2018) and HMMER3 (Mistry et al., 2013).

The *mimp* family of miniature inverted-repeat transposable elements has previously been associated with putative effector genes (Schmidt et al., 2013; van Dam and Rep, 2017; Armitage et al., 2018). Genes were identified within 2 kb of a mimp sequence in the assembled and reference genomes using the consensus sequence for the mimp 3′ inverted repeat (Schmidt et al., 2013) using Perl regular expressions/CAGTGGG..GCAA[TA]AA/ and /TT[TA]TTGC..CCCACTG/.

Searches for homologs of known *FTF*s were performed using tBLASTx against the FON genome. Searches were performed for *SGE1*, a core region transcription factor involved in pathogenicity response, and a further nine transcription factor families (TF1-9), with genes predominantly found on *F. oxysporum* LS regions (Nino-Sanchez et al., 2016; van der Does et al., 2016; Armitage et al., 2018). Sequences used were those detailed in Nino-Sanchez et al. (2016) and Armitage et al. (2018). Genes intersecting the locations of these blast hits were identified using Bedtools v.2.2.6 (Quinlan and Hall, 2010). Coding sequence of these genes was extracted and aligned in Geneious 10.0.3^3^ using the MAFFT algorithm, along with query sequences. A cladogram was generated for TF1 family homologs of *FTF1* and *FTF2* genes in Geneious using a Neighbour joining approach and the consensus tree displayed using the R package GGtree v1.12.4 (Yu et al., 2017).

## Results

### *Fusarium* Isolates From *Narcissus* Vary in Morphology and Virulence

**FIGURE 1:**
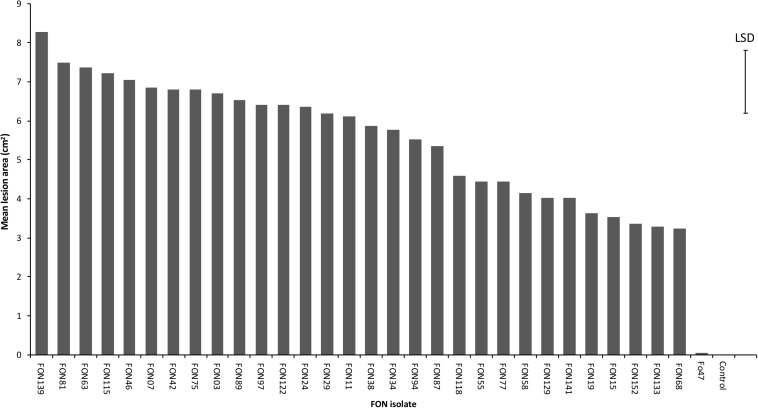
Virulence of 30 *Fusarium oxysporum* isolates against *Narcissus* (cv. Carlton) as measured by mean lesion area at 20 dpi (cm^2^). The error bar indicates the maximum LSD (5% level) following ANOVA. Standard error values ranged from 0.20 to 0.87 with a mean of 0.55.

A total of 154 *Fusarium* isolates were obtained from *Narcissus* bulb samples and classified into eight morphology groups ranging in colour from purple/pink to pale orange/white (Supplementary Table S1 and Supplementary Figure S2). The number of isolates in each group (Table 1) ranged from four in group 3 (purple/red colonies) to 50 in group 4 (white/peach colonies) and this latter group was by far the most common (32.5% of total isolates). Culture morphology was not related to isolate origin and generally, isolates from a single location or *Narcissus* variety comprised of more than one morphology group. The 30 *Fusarium* isolates selected for further analysis comprised group 1, five isolates; group 2, three isolates; group 3, one isolate; group 4, 10 isolates; group 5, one isolate, group 6, two isolates; group 7, seven isolates; group 8, one isolate (Table 1). All isolates were pathogenic on *Narcissus* cv. Carlton but significant differences in virulence were observed (*P* < 0.001, Figure 1 and Table 1) with mean lesion sizes ranging from 3.2 cm^2^ (FON68) to 8.3 cm^2^ (FON139). No lesions were observed on mock inoculated (control) bulbs. The mean lesion size caused by the known non-pathogenic isolate Fo47 was 0.05 cm^2^, which was not significantly different from the control treatment (Figure 1).

### FON Isolates From *Narcissus* Are From a Single Lineage

**FIGURE 2:**
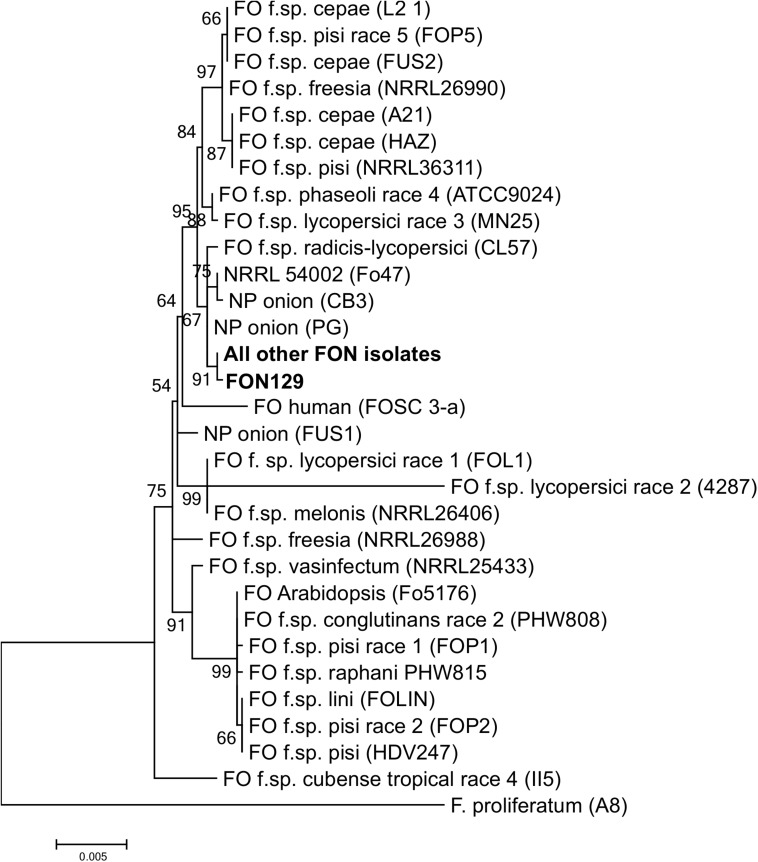
Maximum likelihood tree showing relationships between *Fusarium oxysporum* isolates from *Narcissus* and other hosts based on a concatenated alignment of *β-tubulin*, *translation elongation factor 1-α* and *RNA polymerase II second largest subunit*. Numbers represent bootstrap (1000 replicates) and the scale bar indicates 0.005 substitutions per site. An isolate of *F. proliferatum* was used to root the tree. Additional sequences were obtained from Taylor et al. (2016). NP, non-pathogenic.

BLAST analysis of *EF1-α* sequences identified all 30 selected *Fusarium* isolates as *F. oxysporum* and as they were all pathogenic on *Narcissus* they were designated as FON. Despite the wide variation in morphology, the concatenated sequences of three housekeeping genes (*EF1-α*, *TUB2* and *RPBII*), totalling 2698 bp, revealed that all 30 FON isolates were identical with the exception of isolate FON129 which showed a single base change (T to A) in the *TUB2* region resulting in a single amino acid change from phenylalanine to tyrosine. The ML tree showed that all FON isolates clustered in a single clade that was separated from other *F. oxysporum* f. spp. suggesting they were from a single lineage (Figure 2).

### *SIX* and *FTF* Gene Complements Differ Within FON

The 30 FON isolates contained different complements of *SIX* genes as determined by PCR (Table 1). Three isolates contained *SIX*10 only, one isolate *SIX*9 and *SIX*10, 25 isolates *SIX*7, *SIX*9, *SIX*10 and *SIX*12, with a single isolate (FON63) containing *SIX*7, *SIX*9, *SIX*10, *SIX*12 and *SIX*13. The sequences of the *SIX* gene amplicons were identical for all FON isolates. There were high levels of similarity between FON and FOL *SIX* genes with at least 84% protein identity and 93% nucleotide identity (Supplementary Table S3). In the case of *SIX*9, no amplification was observed for FON using the FOL primers, but products were obtained with the FOC primers; the FON *SIX*9 gene was 99% identical to FOC *SIX*9. Despite the high level of sequence homology, *SIX* gene sequences were unique to FON following BLAST searches. The three putative effector genes identified the genome analysis (BFJ63_5681, BFJ63_18062, BFJ63_18633) were all present in each of the 30 FON isolates.

All 30 FON isolates contained the *FTF2* gene while 24 isolates contained *FTF1* where two different sequence types (A, B) were identified (Table 1). Based on percentage amino acid identity, FON *FTF1a* and *FTF1b* were closely related to the corresponding *FTF1a* and *FTF1b* genes of *F. oxysporum* f. sp. *melonis* (Nino-Sanchez et al., 2016). Analysis of the FON139 assembled genome confirmed the presence of a single copy of both *FTF1* and *FTF2* in this isolate (described below).

### FON *SIX* Genes and Other Putative Effectors Are Expressed *in planta*

**FIGURE 3:**
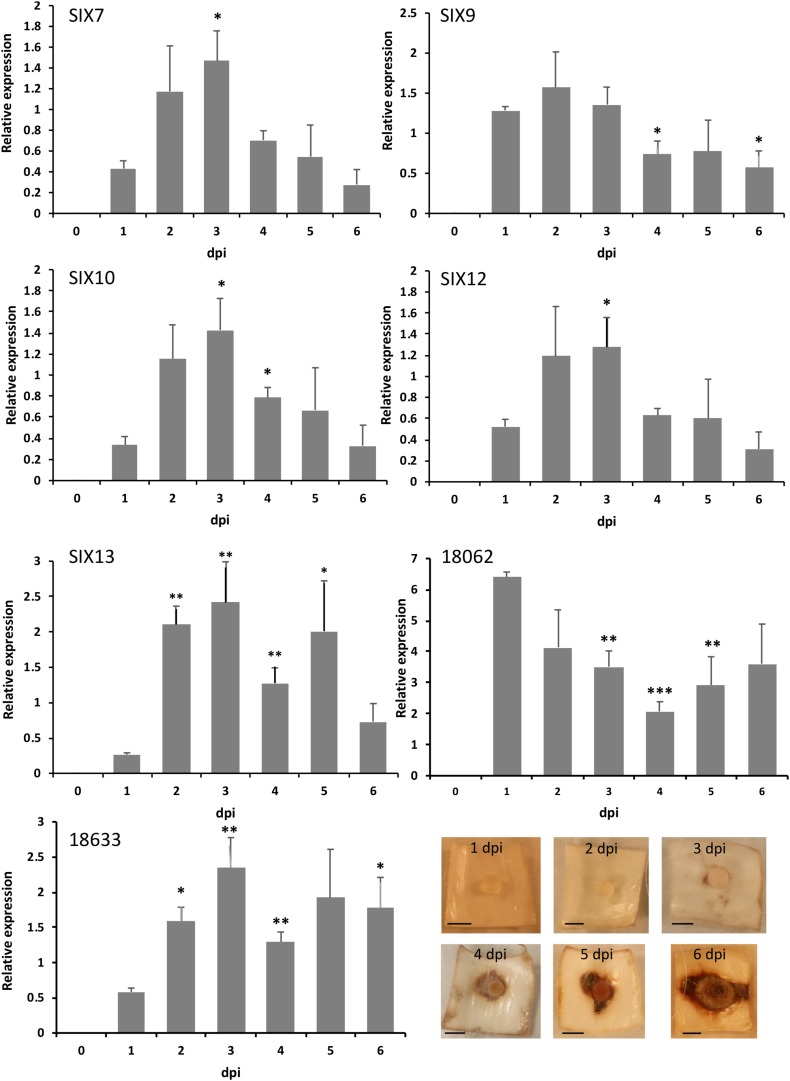
Quantitative expression of *SIX* genes and two other putative effectors *in planta* following inoculation of Narcissus (cv. Carlton) scales with isolate FON63. Expression was calculated relative to the geometric mean of *translation elongation factor 1-α* and *β-tubulin*. Error bars show the standard error of the mean of four biological replicates; dpi, days post-inoculation. Asterisks indicate expression levels significantly different from 1 dpi based on two-tailed *T*-tests (^∗^*P* < 0.05, ^∗∗^*P* < 0.01, ^∗∗∗^*P* < 0.001). Images show the progression of disease symptoms at the time-points used for RNA extractions; scale bars are 0.5 cm.

Isolate FON63 was chosen for expression analyses as it was highly virulent and contained the maximum complement of five *SIX* genes. All *SIX* genes tested were expressed *in planta* and significant upregulation was observed for *SIX*7, *SIX*10, *SIX*12 and *SIX*13 (Figure 3). No upregulation was observed for *SIX*9, but a high level of expression was seen at 1 dpi, possibly suggesting that this gene is upregulated at an earlier time. *SIX* gene expression peaked at 3 dpi when disease symptoms were just starting to develop in the scale tissue, after which expression declined. As for *SIX*9, the putative effector gene BFJ63_18062, showed a very high expression level at 1 dpi while BFJ63_18633 showed a similar expression pattern to the *SIX* genes with significant upregulation and peak of expression at 3 dpi. The putative effector BFJ63_5681 was expressed at levels that were too low for accurate analysis (data not shown).

### FON Genome Sequencing, Assembly and Gene Prediction

Illumina sequencing of FON139 led to generation of 40-fold coverage of the genome, which was assembled to 57.6 Mb in 4358 contigs (>500 bp), with an N50 metric of 159 kb (Table 2). Despite fragmentation in the assembly, homologs were detected for 3684 of 3725 (98.9%) core Sodariomycete genes, comparable to results from previous *F. oxysporum* sequencing projects (Table 2). Repetitive and low complexity regions within the assembly were identified, masking 5.7 Mb (9.88 %) of the genome, similar to the 5.6 Mb (10.54%) masked previously for FOC (Armitage et al., 2018).

Gene prediction performed on the softmasked genome resulted in 20,493 genes, with 19,059 predicted from Braker and a further 1642 additional proteins predicted by CodingQuary in intergenic regions (Table 2). The predicted proteome was found to contain homologs to 3666 of 3725 (98.4%) core Sodariomycete genes, indicating that gene prediction led to the annotation of the majority of FON gene models. The assembly and gene models were deposited at GenBank as a Whole Genome Shotgun project under the accession PRJNA338236. The version described in this paper is the first version, MQTW00000000.

### Orthology Analysis and Identification of Core and Unplaced Regions of the FON Genome

**FIGURE 4:**
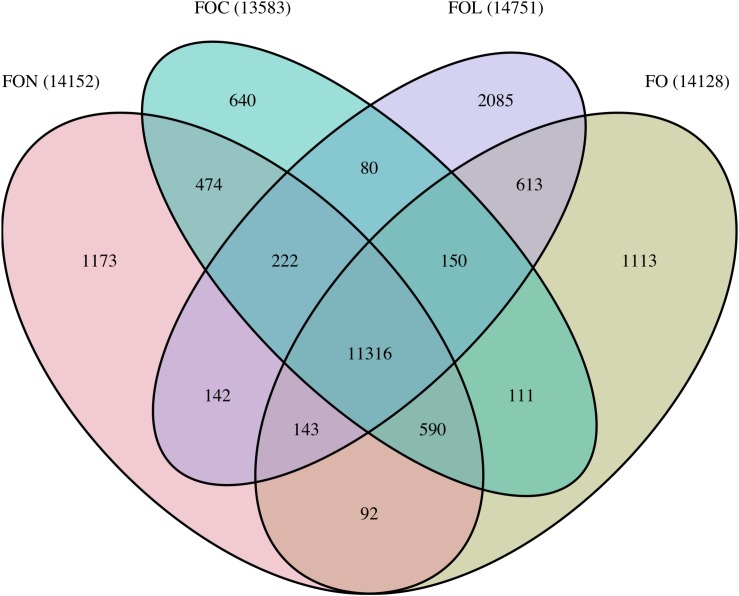
Summary of shared and unique orthogroups between FON, FOC, FOL and FO genomes. Bracketed numbers indicate the total number of orthogroups for each genome.

Orthology analysis clustered 92,237 proteins from FO, FON, FOC and FOL proteomes into 18,944 orthologue groups (Figure 4). Of these, 11,316 orthogroups were common to all isolates, containing 77,393 proteins including 17,147 proteins from FON. The remaining 3554 FON proteins were shared between a subset of isolates or unique to FON. Orthologous genes were used to explore macrosynteny and conservation of effectors, as below.

**FIGURE 5:**
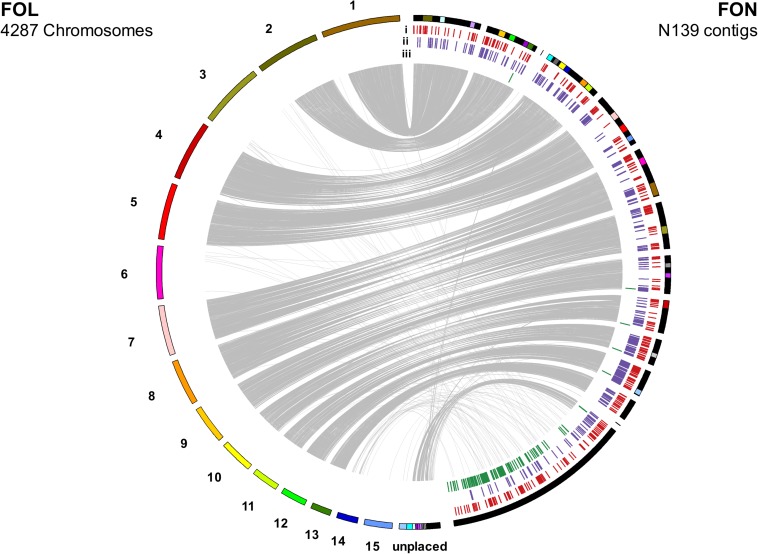
Synteny between FOL and FON genomes as determined by linking single copy orthologous genes. The 14.9 Mb of FON contigs not showing synteny with FON chromosomes are grouped following syntenous contigs. Annotation tracks under FON contigs show distribution of: (i) secreted genes with an effector-like structure, (ii) secreted carbohydrate active enzymes and (iii) *mimps* throughout the genome.

Macrosynteny between FON and FOL assemblies was assessed using 7369 proteins in a 1:1 relationship between FOL and FON. The size of the FON core genome was consistent with that found in other *F. oxysporum* genomes with 273 contigs, equivalent to 42.7 Mb which showed macrosynteny with the 11 FOL core chromosomes (Figure 5). Remaining contigs comprised a total size of 14.9 Mb, representing the putative LS portion of the FON genome.

### *SIX* Genes Are Located Within Putative LS Regions of the FON Genome

BLAST searches identified single homologs of FOL *SIX*7, 9, 10, 12 in putative LS regions of the FON genome. FON homologs of *SIX*7, 10 and 12 were located on a single 7594 bp contig (contig_728; Table 3) whereas *SIX*9 was located on a 2809 bp contig (contig_1485; Table 3). The FON *SIX*9 homolog was a member of the same orthogroup as the *SIX*9 homologs from both FOC and FOL. FON *SIX*7 and *SIX*12 genes were assigned to orthogroups containing their corresponding *SIX*7 and *SIX*12 genes from FOC, but not FOL, reflecting the divergence between these rapidly evolving genes. Similarly, *SIX*10 did not cluster with any other genes. The presence of FON *SIX* gene homologs in LS regions but lack of synteny with FOL and FOC indicates a common origin for these regions but divergence in gene content.

### *Mimps* Are Expanded in the FON Genome and Allow Prediction of Novel Effector Candidates

The FON genome was found to contain over 30% more *mimps* than FOC or FOL genomes, with 207 *mimp* sequences identified (Table 2). These *mimp* sequences were primarily located in putative LS regions of the FON genome, with only six on contigs that showed synteny to FOL chromosomes, four on FON_contig_172 that showed synteny to an unplaced FOL contig DS231739.1 and the remaining 197 on unplaced FON contigs. Despite the increased numbers of *mimps* in FON compared with FOC, only 119 genes were within 2 kb (a similar number to FOC) and of these, only 29 encoded secreted proteins (Table 2).

Putative effectors and pathogenicity factors were identified in the FON genome using EffectorP, CAZY and AntiSMASH (Table 2), with particular focus on genes located on LS contigs and the 29 genes encoding secreted proteins within 2 kb of a *mimp* (Table 3). These 29 genes included homologs of *SIX*9 and *SIX*10, but not *SIX*7 and *SIX*12 (not predicted to be secreted) and included 19 that encoded proteins with a predicted effector-like structure and three encoding secreted CAZYmes. Of the remaining seven genes, annotations included one STAND domain, one alpha/beta hydrolase, one lipase, two peptidases, and two proteins with no recognisable domains (Table 3). Two of the 29 effector candidates were located on FON contigs corresponding to core FOL chromosomes 10 and 11 (BFJ63_3347, BFJ63_5681), with both being members of orthogroups containing a single gene from all isolates, including the non-pathogenic FO. None of the other genes were in orthogroups with a 1:1 relationship between FON and FO, indicating that these represent genes on LS regions.

### Identification of Transcription Factors in the FON Genome

**FIGURE 6:**
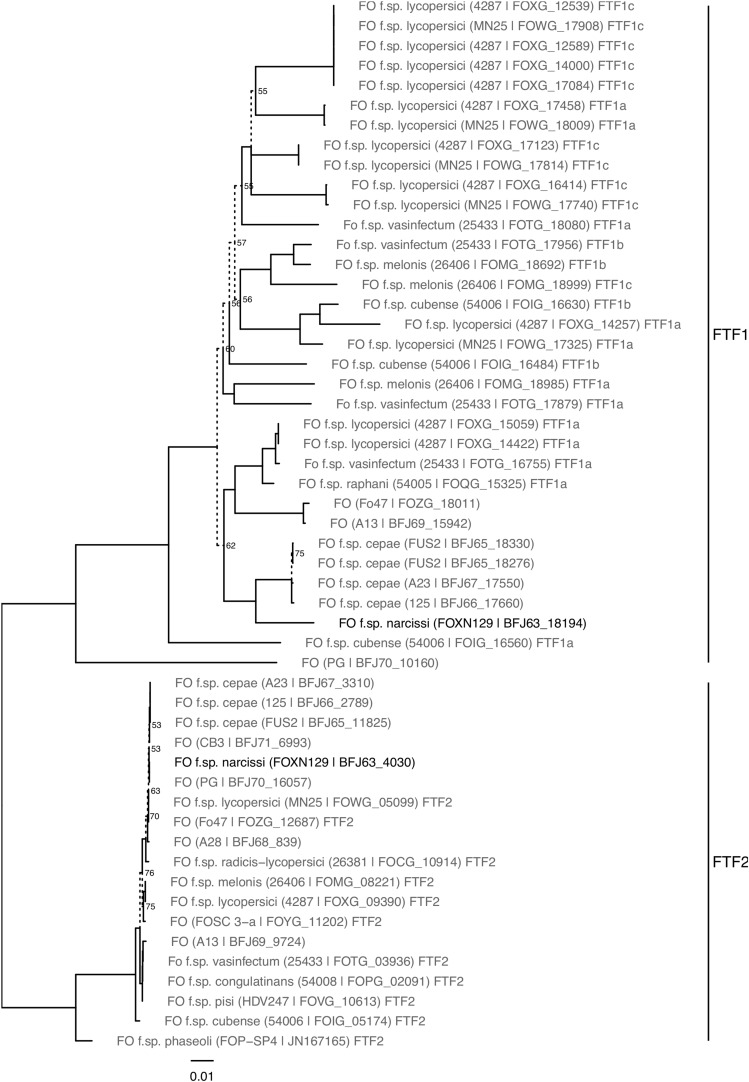
Neighbour joining phylogeny of *FTF* gene sequences from FON and reference sequences. Branches with bootstrap support under 80% from 1000 replicates are marked with dashed lines and labelled with % support values.

Pathogenicity factors within *F. oxysporum* are regulated by genes present on both core and LS regions (van der Does et al., 2016). Orthogroups were identified containing the FOL core genome transcription factor *SGE1* and genes from nine additional FOL transcription factor families (TF1–9), including those from *FOL* LS regions (Table 4). SGE1 had a single FON homolog within the core region of the genome identified as syntenous to FOL chromosome 9. The TF1 family (including *FTF1* and *FTF2*) was identified in FON, with all previously identified FOL and FOC *FTF1* and *FTF2* genes present in a single orthogroup (Orthogroup 472) including two genes from FON. This confirmed the PCR results. Phylogenetic analysis of this orthogroup identified one of these genes as *FTF2* and the other as an *FTF1* homolog distinct from previously sequenced *FTF1* genes (Figure 6). These genes were located on a contig showing synteny to FOL chromosome 9 and a putative LS contig, respectively. Of the eight other transcription factor gene families, seven had homologs in FON, with no homologs found to TF3 genes. In each case, a single homolog was located within contigs syntenous to FOL core chromosomes. In addition to these core genome homologs, additional homologs were identified to TF4, TF6, TF7, TF8 and TF9 genes, all of which were located in putative LS FON contigs (Table 4).

**TABLE 4:** Members of SGE and TF1-9 transcription factor families in FON.

**Transcription factor**	**Function**	**Orthogroup**	**Orthogroup contents**	**FON genes in orthogroup**		
				**FON gene ID**	**FON contig**	**FOL chromosome**
SGE		Orthogroup10386	FON(1):FOL(1):FOC(1):FO(1)	BFJ63_6144	Contig_35	9
TF1 (*FTF2*)	Zn(2)-C6 fungal transcription factor	Orthogroup472	FON(2):FOL(11):FOC(3):FO(2)	BFJ63_4030	Contig_20	9
TF1 (*FTF1*)				BFJ63_18194	Contig_853	–
TF2	Forkhead transcription factor	Orthogroup4647	FON(1):FOL(2):FOC(1):FO(1)	BFJ63_4125	Contig_21	1
TF4	Zn finger containing protein	Orthogroup1633	FON(2):FOL(3):FOC(2):FO(2)	BFJ63_8640	Contig_61	–
				BFJ63_17657	Contig_629	–
TF5	Basic-leucine zipper (bZIP) transcription factor	Orthogroup4960	FON(1):FOL(2):FOC(1):FO(1)	BFJ63_10518	Contig_87	8
TF6	Zn finger containing protein	Orthogroup2432	FON(3):FOL(2):FOC(1):FO(1)	BFJ63_8562	Contig_60	5
				BFJ63_16235	Contig_305	–
				BFJ63_16773	Contig_388	–
TF7	Zn finger containing protein	Orthogroup413	FON(2):FOL(12):FOC(1):FO(5)	BFJ63_13207	Contig_140	9
				BFJ63_16716	Contig_379	–
TF8	Zn(2)-C6 fungal transcription factor	Orthogroup174	FON(5):FOL(21):FOC(2):FO(3)	BFJ63_7317	Contig_46	7
				BFJ63_16714	Contig_379	–
				BFJ63_17643	Contig_624	–
				BFJ63_19029	Contig_1453	–
				BFJ63_19368	Contig_1830	–
TF9	Basic-leucine zipper (bZIP) transcription factor	Orthogroup457	FON(5):FOL(11):FOC(2):FO(1)	BFJ63_8227	Contig_56	5
				BFJ63_16715	Contig_379	–
				BFJ63_18199	Contig_857	–
				BFJ63_18750	Contig_1192	–
				BFJ63_18830	Contig_1255	–

*The contig location in the FON genome is shown for each gene, as well as the corresponding location in the FOL genome, allowing identification of LS genes within these families.*

## Discussion

*Fusarium* was consistently isolated from *Narcissus* bulbs samples with typical basal rot symptoms obtained from different locations and cultivars in the United Kingdom and the collection of over 150 isolates represents a valuable resource. The 30 isolates selected on the basis of culture morphology and origin were all identified as *F. oxysporum* and as they were all pathogenic on *Narcissus* bulbs were designated as FON. This confirms previous research that identified FON as the main causal agent for *Narcissus* basal rot in the United Kingdom (Price, 1975; Beale and Pitt, 1989). While the FON isolates showed considerable variation in morphology and virulence as reported by Linfield (1994) (a common feature of *F. oxysporum* in other pathosystems, Gordon and Martyn, 1997), there was no variation within the three housekeeping genes except a single base change in *TUB2* for isolate FON129. Mutations in this gene have been associated with resistance to benzimidazole fungicides in many fungi including *F. oxysporum* where the same substitution but in a different position has been reported (Chen et al., 2014; Petkar et al., 2017). As the fungicide thiabendazole is extensively used to control FON (Hanks, 2013), it is possible that this mutation in FON129 confers resistance to benzimidazole fungicides but this has yet to be tested. The concatenated phylogenetic tree grouped all FON isolates in a single clade, suggesting a single evolutionary lineage. Similarly, a monophyletic origin has also been reported for FOC and *F. oxysporum* f. sp. *ciceris* (Demers et al., 2014; Taylor et al., 2016), which is in contrast to FOL (Biju et al., 2017), *F. oxysporum* f. sp. *apii* (Epstein et al., 2017) and *F. oxysporum* f. sp. *cubense* (Czislowski et al., 2018) which are polyphyletic. In this latter case, effector genes allowing *F. oxysporum* f. spp. to infect specific hosts are thought to have been gained by horizontal gene transfer (van Dam et al., 2016).

Despite an apparent monophyletic origin for FON, significant variation was observed in the *SIX* gene complement. This is highly unlikely to be due to primer mismatches as these primers are effective on a range of *F. oxysporum* f. spp. (Lievens et al., 2009; Taylor et al., 2016) and genome analysis of isolate FON139 revealed 95–100% primer matches. Two FON isolates contained *SIX*10 only with the majority of isolates containing *SIX*7, *SIX*9, *SIX*10 and *SIX*12. *SIX* genes are consistently associated with pathogenicity of other *F. oxysporum* f. spp., (Edel-Hermann and Lecomte, 2019) and several studies have shown that they are secreted into the xylem sap during infection (Houterman et al., 2007; Ligat et al., 2011; Schmidt et al., 2013; de Sain and Rep, 2015). FON *SIX*9 and *SIX*10 as well as a range of putative novel effectors were predicted to be secreted. Whilst FON *SIX*7 and *SIX*12 were not predicted to be secreted, FOL *SIX*12 lacks a signal peptide yet was isolated from the xylem sap of infected tomato plants (Schmidt et al., 2013) so must be secreted by a different mechanism. Functional studies have shown that knocking out certain *SIX* genes can lead to a reduction in virulence against tomato (Rep, 2005; Houterman et al., 2009; Gawehns et al., 2014; Ma et al., 2015). Similarly, studies on *F. oxysporum* infecting banana and cabbage have also shown that knocking out *SIX* genes directly impacts on virulence, confirming the importance of these genes across multiple *F. oxysporum* f. spp. (Li et al., 2016; Niu et al., 2016; Widinugraheni et al., 2018). Therefore, it is highly likely that *SIX* genes are involved in pathogenicity against *Narcissus* though gene knockouts would be required to definitively prove this. In FOL, at least four SIX genes (SIX 1, 3, 4 and 5) are recognised by host resistance genes (Rep et al., 2004; Houterman et al., 2008, 2009; Takken and Rep, 2010; Ma et al., 2015) and in the future, it may be possible to identifiy resistance genes in Narcissus which recognise FON SIX genes.

Previously, we identified a single FOC isolate which exhibited reduced virulence and contained only two of the seven FOC *SIX* genes (Taylor et al., 2016), suggesting that multiple *SIX* genes contribute to full virulence. In FON, the second most pathogenic isolate (FON81) only contained *SIX*10 while conversely, the least pathogenic isolate (FON68) contained four *SIX* genes. This suggests that there may be considerable functional redundancy in known *SIX* genes in FON. This is not unprecedented as FOL race 2 emerged through loss of *SIX*4 in order to avoid detection by a corresponding resistance gene introgressed into tomato (Houterman et al., 2008; Takken and Rep, 2010). New races can also emerge through mutation of a *SIX* gene, as for FOL race 3 where a single base change in the *SIX*3 gene resulted in resistance breakdown through avoidance of detection by another resistance gene (Houterman et al., 2009). Similarly, races of *F. oxysporum* f. sp. *cubense* can be separated by differences in *SIX* gene complements and sequence variations (Meldrum et al., 2012; Fraser-Smith et al., 2014). Past studies have identified cv. St. Keverne as resistant to basal rot (Nicholson et al., 1989; Bowes et al., 1992) while subsequent anecdotal evidence has indicated resistance breakdown (Hanks, 1996), supporting the theory that different races of FON may have evolved. However, the genetic basis for FON resistance in *Narcissus* has not been determined and further work is required to assess whether there is variation in response of *Narcissus* cultivars from different genetic backgrounds to isolates of FON with differing *SIX* gene profiles. FON15 was isolated from an infected St. Keverne bulb but showed no evidence of effector gene mutations although a whole genome sequence of this isolate would be required to definitively address this question.

All five *SIX* genes identified in FON63, as well as two putative novel effectors, were expressed *in planta* and significant upregulation was observed for *SIX*7, 10, 12 and 13. The upregulation of FON *SIX* genes coupled with the very high level of *SIX* gene sequence conservation between FOL and FON strongly suggests a conserved function, supporting the hypothesis that these genes are effectors in FON. As noted above, FON isolates containing only *SIX*10 were still highly virulent. Therefore, it is likely that other putative effectors, as identified in the FON genome analysis, are involved in pathogenicity and that there is redundancy in *SIX* gene function. Genome analysis identified 119 FON genes within 2 kb of a *mimp* sequence of which 29 encoded secreted proteins, representing further effector candidates. *Mimp* sequences have been shown to be present in the core genome of other *F. oxysporum* f. sp. and present in *F. proliferatum* and *Fusarium hostae* indicating that their presence is not unique to LS regions (van Dam and Rep, 2017; Armitage et al., 2019). The lack of orthologous genes in the non-pathogenic FO isolate fo47 gene models supports the LS status of 19 of these genes. The LS gene BFJ63_18633 (and potentially the LS gene BFJ63_18062) showed significant upregulation over time *in planta*, strongly suggesting a role for these genes in virulence on *Narcissus*. Lack of quantifiable expression for BFJ63_5681, located in the core region of the FON genome, questions whether effector candidates in the vicinity of *mimps* have the same association with pathogenicity when located in the core genome. Whilst this study focuses on the role of effector genes in pathogenicity, it should be noted that other mechanisms of pathogenicity may also be important. For example, production of toxins such as Fusaric acid has been shown to directly impact pathogenicity in *F. oxysporum* (López-Díaz et al., 2018).

The molecular characterisation of FON presented here provides potential targets for distinguishing this pathogen from other *F. oxysporum* f. spp. which has previously been very challenging due to the polyphyletic nature of the *F. oxysporum* complex (O’Donnell et al., 1998; Baayen et al., 2000). Previous work indicated that *F. oxysporum* f. spp. *narcissi* and *dianthi* could not be separated based on *SIX* gene complement/sequence (Taylor et al., 2016) but we have subsequently discovered that this historic isolate from the Warwick collection was mislabelled as it is actually pathogenic on *Narcissus* (Taylor et al., unpublished) and hence is now identified as FON. Whilst *EF1-α* sequences could be used as a preliminary tool to identify FON, sequence differences from other *F. oxysporum* f. spp. are not sufficient for molecular diagnostics. FON *SIX*10 is one potential target for a diagnostic locus but has a high level of sequence identity with FOC *SIX*10 (96% nucleotide identity). The newly identified putative effectors from the FON genome analysis therefore may represent more suitable diagnostic targets.

Genome sequencing at 40× coverage led to a FON assembly containing over 98% of expected Sodariomycete gene models. Despite fragmentation, synteny was identified between FON contigs and FOL chromosomes, allowing identification of genes in both core and LS regions of the genome. In total, putative LS regions represented 14.9 Mb, falling within the 4–19 Mb range of LS regions identified in other *F. oxysporum* f. spp. (Schmidt et al., 2016; van Dam et al., 2016; Williams et al., 2016). Transposons have been shown to play an important role in adaptation of fungal pathogens, driving genomic rearrangement (Faino et al., 2016). The genome of FON139 possessed 30% more *mimps* than identified in FOL or FOC, with the majority of these located in unplaced FON contigs. This number may reflect a conservative estimate of *mimps* in this genome as assemblies generated from Illumina sequence data have been shown to underestimate *mimps* in comparison to PacBio sequence data (van Dam and Rep, 2017). Despite this, FON carried similar numbers of *mimp*-associated secreted proteins to FOC and FOL. All *SIX* gene homologs were located within 2 kb of a *mimp*. However, *mimp* sequences are not determinants of gene regulation (Schmidt et al., 2013) and may play other roles in pathogen adaptation and evolution. Other miniature inverted-repeat transposable elements have been shown to be mobilised by full-length class II transposable elements (Feschotte et al., 2002; Dufresne et al., 2007; Bergemann et al., 2008) and impala sequences have been shown to be active in some *F. oxysporum* strains (Hua-Van et al., 2001). As there is diversity in effector gene complements between FON isolates and *mimps* are abundant, FON may be an important pathogen for studying their role in genomic rearrangement within and between dynamic LS regions. Pertinent questions include whether *mimps* are still active in FON, whether *mimps* are driving race evolution and whether LS regions are undergoing more reshuffling in FON.

Homologs to genes in transcription factor families known to regulate expression of virulence genes in *F. oxysporum* f. spp. were also identified in FON. FON139 carried a single homolog on the core genome to *SGE* and each of TF1-9 except TF3, congruent with the original description of these genes in FOL (van der Does et al., 2016). Additional genes in TF families were identified in unplaced FON regions likely represent LS transcription factors. TF gene families in FON showed a different pattern of expansion from those observed in FOL; TF2 and TF5 had an additional copy in FOL LS regions, but there were no additional copies in FON. In contrast, TF6 had a single copy in a FOL LS region, while FON contained two additional copies in unplaced contigs. Expansion of these TF1-9 families onto LS regions has been hypothesised to confer selective advantage through dosage effect, with more copies of TF1 being associated with greater virulence in *F. oxysporum* f. sp. *phaseoli* (de Vega-Bartol et al., 2010; van der Does et al., 2016). As such, genes regulated by these TFs may be of differing importance in FOL and FON host infection. Occurrence of the TF1 LS gene *FTF1* was determined within the 30 FON isolates, leading to the identification of two distinct *FTF1* haplotypes. Genes from this family have been shown to regulate *SIX* gene expression and contribute to virulence (Nino-Sanchez et al., 2016). This may also be the case in FON, although further work is required to understand how many genes are present in this family in different FON isolates. It may be that where a negative PCR was observed (6 out of 30), these isolates have an alternate, divergent copy of *FTF1*. The FON *FTF2* homologue was present in all isolates and has been shown to exist in a wide range of ascomycetes (Nino-Sanchez et al., 2016).

This is the first study to characterise molecular variation in FON and provide an analysis of the FON genome. Identification of expressed genes potentially associated with virulence provides the basis for future functional studies and new targets for molecular diagnostics.

## Data Availability Statement

The raw data supporting the conclusions of this article will be made available by the authors, without undue reservation, to any qualified researcher. GenBank Accession Numbers: MQTW01000000 – FON139 genome assembly; MN078955 – FON EF1α sequence; MN078956 – FON RPB2 sequence; MN078957 – FON129 TUB2 sequence; MN078958 – FON TUB2 sequence; MN078959 – FON *FTF1*a sequence; MN078960 – FON *FTF1*b sequence; MN078961 – FON *SIX*7 sequence; MN078962 – FON *SIX*9 sequence; MN078963 – FON *SIX*10 sequence; MN078964 – FON *SIX*12 sequence; MN078965 – FON63 *SIX*13 sequence.

## Author Contributions

AT planned and carried out the experiments, analysed the data, created figures, and drafted and edited the manuscript. AA carried out the FON genome analysis and all bioinformatics, created figures, and drafted and edited the manuscript. CH and MH carried out the experiments. AJ carried out the experiments and edited the manuscript. RH obtained funding, planned experiments, and edited the manuscript. JC obtained funding, planned experiments, and wrote and edited the manuscript.

## Conflict of Interest

The authors declare that the research was conducted in the absence of any commercial or financial relationships that could be construed as a potential conflict of interest.

## Footnotes

1 http://www.repeatmasker.org

2 http://transposonpsi.sourceforge.net

3 https://www.geneious.com
