# Supplementary figures and images for: Basal Rot of Narcissus: Understanding Pathogenicity in Fusarium oxysporum f. sp. narcissi
Source: Front Microbiol. 2019 Dec 19;10:2905. doi: 10.3389/fmicb.2019.02905 (PMC6930931; doi:10.3389/fmicb.2019.02905)

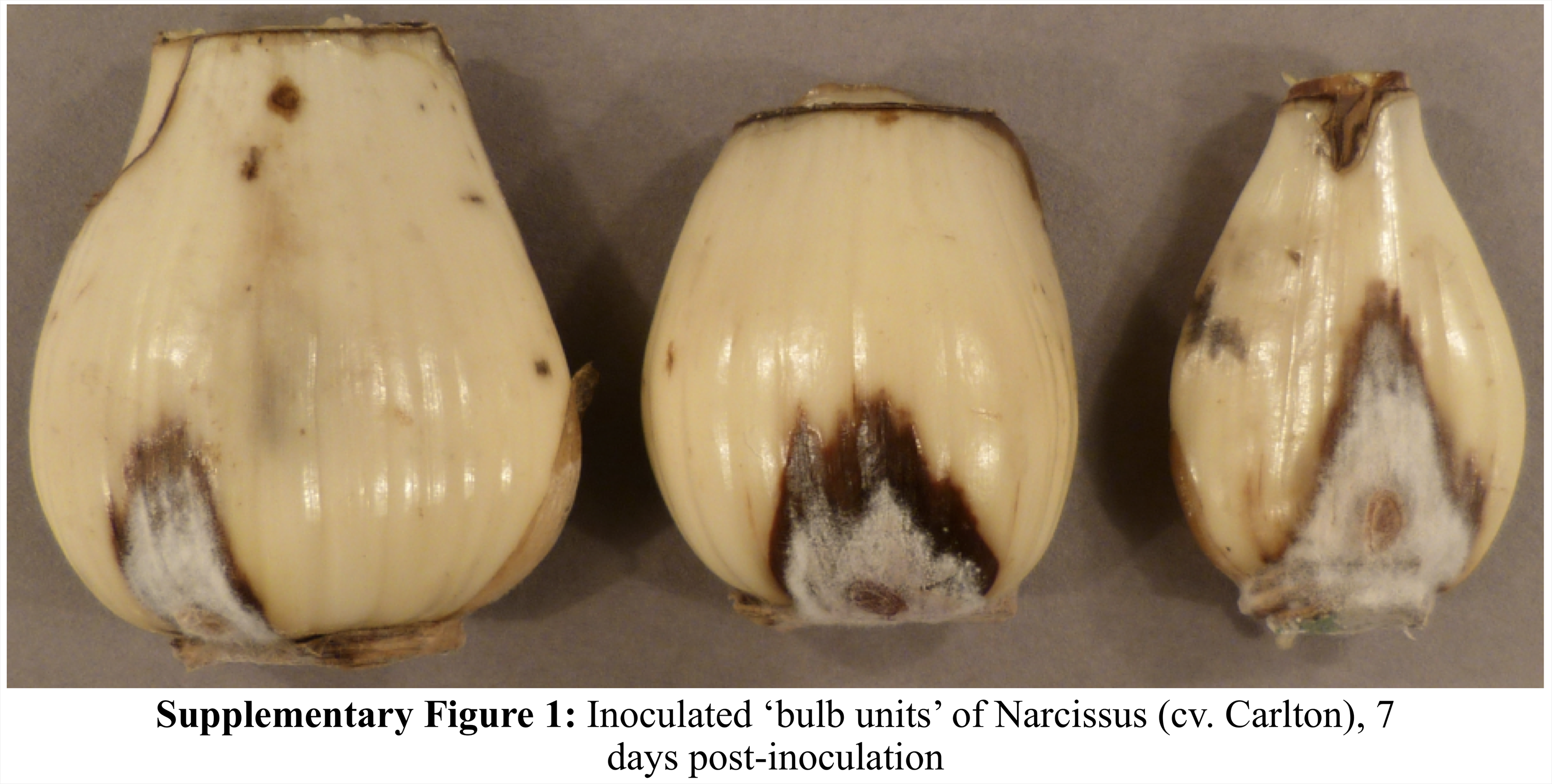

Supplement: Supplementary file 1 [file Image_1.TIF]

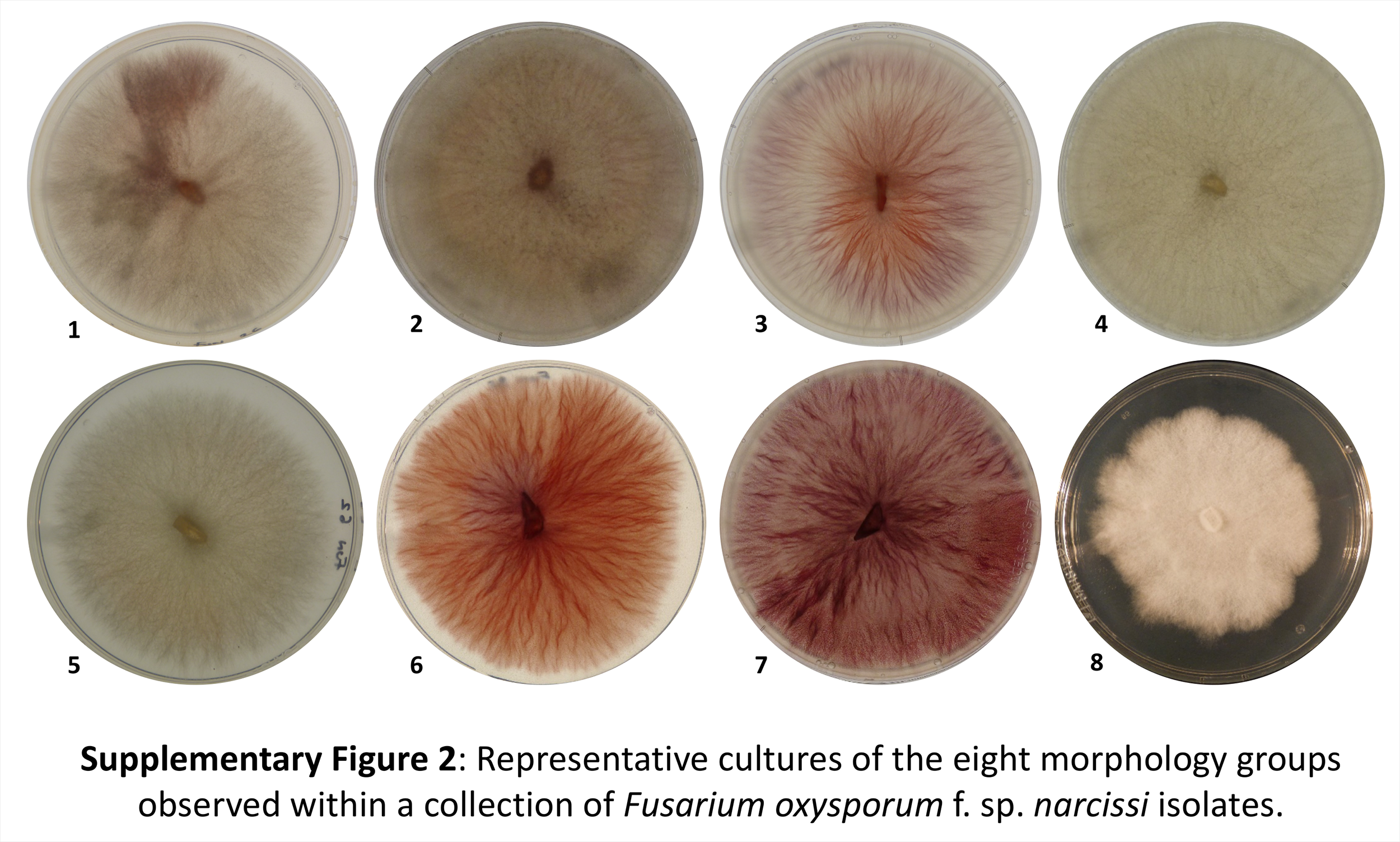

Supplement: Supplementary file 2 [file Image_2.TIF]
