# Supplementary material for: Basal Rot of Narcissus: Understanding Pathogenicity in Fusarium oxysporum f. sp. narcissi
Source: Front Microbiol. 2019 Dec 19;10:2905. doi: 10.3389/fmicb.2019.02905 (PMC6930931; doi:10.3389/fmicb.2019.02905)
Supplement: Supplementary file 3 [file Table_1.DOCX]

**Supplementary Table 1.** Number of *Fusarium* isolates assigned to eight morphology groups from different *Narcissus* bulb sources and locations. Morphology groups: 1 = purple; 2 = purple, concentric rings; 3 = purple, red in middle; 4 = pale white top, peach tinge on reverse; 5 = pale white top, orange/pink tinge on reverse; 6 = red flat; 7 = purple/pink, 8 = white.

|  |  | **No. of isolates in morphology group** | | | | | | | |
| --- | --- | --- | --- | --- | --- | --- | --- | --- | --- |
| **Location** | **Cultivar** | **1** | **2** | **3** | **4** | **5** | **6** | **7** | **8** |
| Norfolk | White Lion | 0 | 1 | 0 | 3 | 0 | 0 | 2 | 0 |
| W Cornwall | Carlton | 0 | 0 | 0 | 1 | 0 | 0 | 3 | 0 |
| E Cornwall | Carlton | 0 | 2 | 0 | 0 | 0 | 0 | 2 | 0 |
| Spalding, Lincs | St Keverne | 0 | 1 | 0 | 1 | 0 | 1 | 1 | 0 |
| Boston, Lincs | Quirinus | 1 | 0 | 0 | 2 | 0 | 0 | 0 | 0 |
| Boston, Lincs | Carlton | 0 | 1 | 2 | 0 | 0 | 0 | 1 | 0 |
| Norfolk | Fortune | 1 | 0 | 1 | 0 | 0 | 0 | 1 | 2 |
| Holt, Norfolk | Sempre Avanti | 0 | 0 | 0 | 5 | 0 | 0 | 0 | 0 |
| Spalding, Lincs | Carlton | 1 | 3 | 0 | 0 | 0 | 0 | 0 | 0 |
| Holt, Norfolk | Golden Ducat | 3 | 2 | 0 | 0 | 0 | 0 | 0 | 0 |
| Truro, Cornwall | Salome | 0 | 0 | 0 | 4 | 0 | 0 | 0 | 0 |
| Penzance, Cornwall | Apotheose | 0 | 2 | 0 | 2 | 0 | 0 | 0 | 0 |
| Penzance, Cornwall | White Lion | 0 | 0 | 0 | 1 | 0 | 0 | 2 | 0 |
| Spalding, Lincs | Great Leap | 0 | 2 | 0 | 2 | 0 | 0 | 1 | 0 |
| Truro, Cornwall | Magnificence | 0 | 0 | 0 | 0 | 3 | 0 | 0 | 0 |
| Spalding, Lincs | Spellbinder | 0 | 1 | 1 | 0 | 0 | 1 | 1 | 0 |
| Holt, Norfolk | Golden Ducat | 1 | 0 | 0 | 2 | 0 | 0 | 1 | 1 |
| Norwich, Norfolk | Pheasant's Eye | 1 | 0 | 0 | 3 | 0 | 0 | 1 | 0 |
| Truro, Cornwall | Salome | 0 | 0 | 0 | 5 | 0 | 0 | 0 | 0 |
| Cornwall | Golden Ducat | 0 | 0 | 0 | 0 | 0 | 2 | 1 | 0 |
| Cornwall | Jedna | 0 | 1 | 0 | 3 | 0 | 0 | 0 | 1 |
| Cornwall | Hollywood | 0 | 1 | 0 | 1 | 0 | 0 | 3 | 0 |
| Cornwall | Grenoble | 1 | 0 | 0 | 1 | 1 | 0 | 1 | 0 |
| Cornwall | Mando | 1 | 0 | 0 | 1 | 0 | 0 | 1 | 0 |
| Cornwall | Standard Value | 0 | 0 | 0 | 0 | 1 | 0 | 0 | 1 |
| Falmouth, Cornwall | Welcome | 0 | 0 | 0 | 0 | 1 | 0 | 0 | 1 |
| Falmouth, Cornwall | Copper Court | 2 | 0 | 0 | 0 | 0 | 1 | 2 | 0 |
| Falmouth, Cornwall | Pinza | 0 | 0 | 0 | 2 | 0 | 1 | 0 | 0 |
| Falmouth, Cornwall | Orkney | 2 | 0 | 0 | 0 | 0 | 0 | 0 | 0 |
| Falmouth, Cornwall | Unique | 2 | 0 | 0 | 1 | 0 | 0 | 2 | 0 |
| Falmouth, Cornwall | Queen Mum | 0 | 0 | 0 | 1 | 0 | 0 | 1 | 0 |
| Falmouth, Cornwall | Whiskey Galore | 1 | 0 | 0 | 1 | 0 | 2 | 0 | 0 |
| Falmouth, Cornwall | Hampton Court | 1 | 0 | 0 | 0 | 0 | 1 | 3 | 0 |
| Falmouth, Cornwall | Hambledon | 2 | 0 | 0 | 1 | 0 | 1 | 0 | 0 |
| Falmouth, Cornwall | Scrumpy | 0 | 0 | 0 | 2 | 0 | 0 | 0 | 0 |
| Falmouth, Cornwall | Mithrel | 1 | 0 | 0 | 1 | 0 | 0 | 0 | 0 |
| Falmouth, Cornwall | Silent Valley | 2 | 0 | 0 | 0 | 0 | 0 | 2 | 0 |
| Spalding, Lincs | St Keverne | 2 | 0 | 0 | 0 | 0 | 0 | 3 | 1 |
| Moulton, Lincs | Carlton | 0 | 0 | 0 | 4 | 0 | 0 | 0 | 0 |
| **Total isolates** | **154** | **25** | **17** | **4** | **50** | **6** | **10** | **35** | **7** |
| **% of total isolates** |  | 16.2 | 11.0 | 2.6 | 32.5 | 3.9 | 6.5 | 22.7 | 4.5 |
