# Supplementary material for: Basal Rot of Narcissus: Understanding Pathogenicity in Fusarium oxysporum f. sp. narcissi
Source: Front Microbiol. 2019 Dec 19;10:2905. doi: 10.3389/fmicb.2019.02905 (PMC6930931; doi:10.3389/fmicb.2019.02905)
Supplement: Supplementary file 4 [file Table_2.DOCX]

**Supplementary Table 2**: Primers used for the molecular characterisation of *Fusarium* *oxysporum* f. sp *narcissi* isolates, amplicon sizes and annealing temperatures (adapted from Taylor et al., 2016).

| **Primers** | **Gene** | **Sequences (5’- 3’)** | **Annealing temperature (°C)** |
| --- | --- | --- | --- |
| T1/T22 | *TUB2* | ^1^AACATGCGTGAGATTGTAAGT/  TCTGGATGTTGTTGGGAATCC | 60 |
| 7cF/11aR | *RPB2* | ^1^ATGGGYAARCAAGCYATGGG/  GCRTGGATCTTRTCRTCSACC | 57 |
| exTEF-F/ FUexTEF-R | *EF-1α* | ^1^ACCCGGTTCAAGCATCCGATCTGCGA/ AGCTTGCCRGACTTGATCTCACGCTC | 64 |
| SIX1 | *SIX1* | GTATCCCTCCGGATTTTGAGC/ AATAGAGCCTGCAAAGCATG | 59 |
| SIX2 | *SIX2* | CAACGCCGTTTGAATAAGCA/ TCTATCCGCTTTCTTCTCTC | 59 |
| SIX3 | *SIX3* | CCAGCCAGAAGGCCAGTTT/ GGCAATTAACCACTCTGCC | 59 |
| SIX4 | *SIX4* | TCAGGCTTCACTTAGCATAC/ GCCGACCGAAAAACCCTAA | 59 |
| SIX5 | *SIX5* | ACACGCTCTACTACTCTTCA/ GAAAACCTCAACGCGGCAAA | 59 |
| SIX6 | *SIX6* | CTCTCCTGAACCATCAACTT/ CAAGACCAGGTGTAGGCATT | 59 |
| SIX7 | *SIX7* | ^1^CATCTTTTCGCCGACTTGGT/ CTTAGCACCCTTGAGTAACT | 59 |
| SIX8 | *SIX8* | TCGCCTGCATAACAGGTGCCG/ TTGTGTAGAAACTGGACAGTCGATGC | 59 |
| FOL SIX9 | *SIX9* | GGGTGGACCATATCACGATGTTCG/ GAATACCTGAGTGGAGTTGTGTCTTG | 69 |
| FOC SIX9 | *SIX9* | ^1^GGCCCAGCCCTAGTCTAACTCC/ AACTTAACATGCTGGCCGTCAATCG | 67 |
| SIX10 | *SIX10* | ^1^GTTAGCAACTGCGAGACACTAGAA/ AGCAACTTCCTTCCTCTTACTAGC | 65 |
| SIX11 | *SIX11* | ATTCCGGCTTCGGGTCTCGTTTAC/ GAGAGCCTTTTTGGTTGATTGTAT | 61 |
| SIX12 | *SIX12* | ^1^CTAACGAAGTGAAAAGAAGTCCTC/ GCCTCGCTGGCAAGTATTTGTT | 61 |
| SIX13 | *SIX13* | ^1^CCTTCATCATCGACAGTACAACG/ ATCAAACCCGTAACTCAGCTCC | 61 |
| FOL SIX14 | *SIX14* | ATAAAGTGCGACTGGACTTCTGCC/ ACCCCCATCCACATTCCTAAGCGA | 67 |
| FOC SIX14 | *SIX14* | ACAACACCGCGACGCTAAAAAT/ GCACACTCAGTGCGACAAGTTC | 61 |
| QSIX7 | *SIX7* | Tcgatctctttccaagacaagggca/  gtggacgcggcgttggtgaac | 63 |
| QSIX9 | *SIX9* | GCCGACCCAGACCTACGCTTT/ GCTGGTTTTGGAAGCCCAGTTGT | 63 |
| QSIX10 | *SIX10* | CCCGGAAAGCCTGCATCGACTA/ AGAACAAACGTCGGTGGGACCA | 63 |
| QSIX12 | *SIX12* | TGCTGCTCCAAGTACAAACTACCTT/  GCTGATACCTTTGGGTCCAACGC | 63 |
| FON QSIX13 | *SIX13* | ACAGCACGGGACAGCTTACA-3’ / 5’-CGTCAGAGGGGTAGCCACAT | 60 |
| QTEF | *EF-1α* | Ggtcaggtcggtgctggttacg/  tggatctcggcgaacttgcagg | 63 |
| QTUB | *TUB2* | TTCTGCTGTCATGTCCGGTGT/ TCAGAGGAGCAAAGCCAACCA | 63 |
| FON FTF1 | *FTF1* | ^1^GGGTTGAATCTCACGTATCCTGC/ TCCATTCGAGCCCTGCCCAAAG | 65 |
| FON FTF2 | *FTF2* | CGGTCAAGCAATTCGCATGGC/  GTTCTCTGTCTTGTGGACGTCG | 63 |
| FON18062 | BFJ63_18062 | ATGGCGAACTGGTCTTGGCTCC/ CTTGAGCACCCCACGGAACACT | 65 |
| FON5681 | BFJ63_5681 | CCGTGTTCCTTGCTTTTACTGCCG/ TGCAGCCGCCATCCTTGTAGTAC | 65 |
| FON18633 | BFJ63_18633 | CCAGGCTTTTCCTCGAACCGCA/ TCGACGCTGTAGTCCGCAAAGAG | 65 |

^1^Primer used for sequencing
