# Supplementary material for: Basal Rot of Narcissus: Understanding Pathogenicity in Fusarium oxysporum f. sp. narcissi
Source: Front Microbiol. 2019 Dec 19;10:2905. doi: 10.3389/fmicb.2019.02905 (PMC6930931; doi:10.3389/fmicb.2019.02905)
Supplement: Supplementary file 5 [file Table_3.DOCX]

**Supplementary Table 3.** SIX and FTF genes in *F. oxysporum f.sp. narcissi* with associated nucleotide and protein percentage identities using BLAST (Boratyn *et al*., 2013) in comparison with *F. oxysporum* f.sp. *lycopersici* and *F. oxysporum* f.sp. *melonis* respectively unless otherwise stated.

| **Gene** | **Nucleotide identity (%)** | **Protein identity (%)** |
| --- | --- | --- |
| *SIX7* | 93 | 84 |
| *SIX9* | 99* | 99* |
| *SIX10* | 95 | 87 |
| *SIX12* | 95 | 92 |
| *SIX13* | 94 | 84 |

| **Gene** | **FON FTF1a** | **FON FTF1b** | **FON FTF2** |
| --- | --- | --- | --- |
| *FTF2* | 85.5 | 90.5 | **100** |
| *FTF1a* | **90.0** | 91.7 | 89.3 |
| *FTF1b* | 89.1 | **92.8** | 89.9 |
| *FTF1c* | 89.5 | 91.4 | 88.1 |

*The FON SIX9 gene was compared with FOC due to large divergence from FOL
